# Perfusion-only imaging in pregnant women: A comparative reader study with implications for practice patterns

**DOI:** 10.1097/MD.0000000000030800

**Published:** 2022-09-30

**Authors:** Jennifer A. Schroeder, Quy Cao, Vlasios S. Sotirchos, Jennifer A. Gillman, Thomas Anderson, Stamatoula Pilati, Jacob G. Dubroff, Michael Farwell, Andrew Kozlov, Katrina Korhonen, Daniel A. Pryma, Austin R. Pantel

**Affiliations:** a Department of Radiology, Nuclear Medicine, Atrium Health Wake Forest Baptist Medical Center, Winston-Salem, NC, USA; b Department of Biostatistics, Epidemiology, & Informatics, University of Pennsylvania, Philadelphia, PA, USA; c Interventional Radiology, Memorial Sloan Kettering Cancer Center, New York, NY, USA; d Department of Radiology, Mid-Atlantic Permanente Medical Group, Rockville, MD, USA; e Department of Radiology, University of New Mexico, Albuquerque, NM, USA; f Department of Radiology and Nuclear Medicine, Cook County Health, Chicago, IL, USA; g Department of Radiology, Nuclear Medicine, University of Pennsylvania, Philadelphia, PA, USA; h Radiology Associates of Florida & the University of South Florida, Morsani College of Medicine, Philadelphia, PA, USA; i Radiology Partners, Houston, TX, USA.

**Keywords:** pregnancy, pulmonary embolism, ventilation perfusion stratigraphy

## Abstract

This study seeks to understand the value of ventilation imaging in pregnant patients imaged for suspected pulmonary embolism (PE). Ventilation-perfusion (VQ) scans in this high-risk population were compared to ventilation-only scans. We hypothesize that in this relatively healthy population, the exclusion of ventilation scans will not impact the rate of scans interpreted as positive. This retrospective blinded comparative reader study on collated VQ scans performed on pregnant patients in the course of routine clinical care in *a* > 5 year period (03/2012 to 07/2017). Each set of VQ and perfusion only (Q) studies were reviewed by 8 readers (4 nuclear radiology fellows and 4 nuclear medicine faculty) in random order; the Q scans simply omitted the ventilation images. Readers recorded each study as PE, no PE, or non-diagnostic (prospective investigative study of acute PE diagnosis classifications). Logistic mixed effects models were used to test the association between scan type (VQ vs Q). 203 pairs of studies in 197 patients were included (6 patients had 2 scans). Subjects ranged from 14 to 45 years of age, with a median 28 years. A significant association between scan type and positive/negative classification. Q-scans received more positive classifications than VQ-scans (median of 7.6% vs 6.7%). No association was seen between scan type and positive/indeterminate classification, nor between scan type and negative/indeterminate classification. The exclusion of ventilation images in VQ-scans was associated with a higher rate of positive studies, but this difference was small (<1%). Given the overwhelmingly normal percentage of Q-exams (>90% in our study), and the benefits of omitting ventilation imaging, perfusion-only imaging should be considered a reasonable option for imaging the pregnant patient to exclude PE.

## 1. Introduction

Since the 1960s, pulmonary perfusion nuclear imaging with ^99m^Technetium-macroaggregated albumin (^99m^Tc-MAA) has been extensively used for the evaluation for acute pulmonary embolism (PE).^[1]^ By pairing ^99m^Tc-MAA perfusion imaging with ventilation imaging, acute PE is diagnosed by identifying an area of absent perfusion, but preserved ventilation. Several different ventilation agents have been studied for this application.^[2,3]^ Additionally, multiple studies have examined perfusion-only PE imaging and specific interpretation criteria have been proposed.^[4,5]^ For example, the prospective investigative study of acute PE diagnosis criteria demonstrated that by combining perfusion imaging with a chest radiograph (CXR), the diagnostic accuracy of perfusion-only scintigraphy was similar to computed tomography (CT) of the pulmonary artery (CTPA) and similar to combined ventilation-perfusion (VQ) imaging. Similarly, the modified prospective investigation of pulmonary embolism (PIOPED II) criteria has been shown to perform equivalently to protocols that include ventilation.^[4,5]^ Despite significant benefits to perfusion-only scintigraphy – including less radiation to breast tissue (which is more radiosensitive during pregnancy/lactation) and lower costs^[6]^ – there remains a lack of consensus to the necessity of ventilation-phase imaging. Ultimately, without a guiding recommendation from governing societies, ventilation imaging has largely continued; modified perfusion-only criteria is less commonly used, typically relied upon when technical difficulties preclude diagnostic quality ventilation scintigraphy.

The onset of a global pandemic by COVID-19, forced nuclear imaging communities around the world to question the necessity of the ventilation imaging. Indeed, the society of nuclear medicine and molecular imaging on March 19, 2020 endorsed perfusion-only imaging citing the elevated risk placed on the providers performing ventilation imaging, noting the utility of such studies given the similarities of symptoms between COVID-19 and PE (such as shortness of breath, tachycardia, and chest pain).^[7]^ While there have been commentaries on both the necessity and the dispensable nature of ventilation, the implications of this decision – one based on necessity given the unusual circumstances and not particularly data-driven – have not fully been explored.^[8,9]^ Specifically, the performance of a perfusion-only protocol has not been rigorously compared to a protocol with ventilation. Moreover, such a comparison has not been performed in the setting of a *high-risk population who have been historically excluded from such studies: pregnant women*.

In this reader study, we examine the effect on study performance of omitting ventilation images in planar pulmonary perfusion scans obtained in pregnant patients, a high-risk population with a paucity of prior data. We hypothesize that perfusion-only imaging may lead to more positive studies given the inability to detect matched defects in the absence of ventilation images. However, in this population of pregnant patients with overwhelmingly normal ventilation scans, such a difference may be negligible.

## 2. Methods

### 2.1. Case selection/interpretation procedure

**Figure 1.**
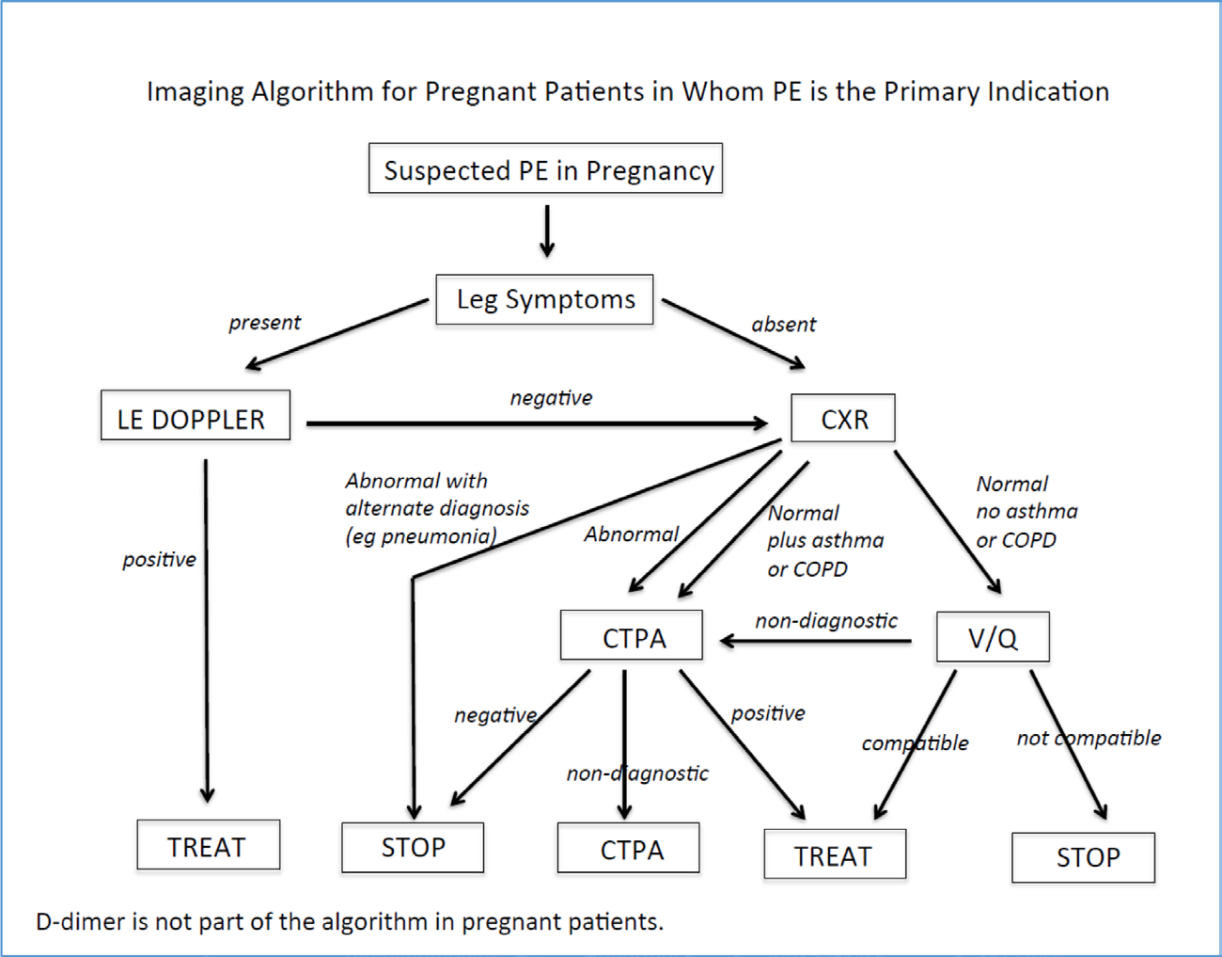
Imaging algorithm used to screen pregnant patients suspected to have pulmonary embolism. Sourced through the Research Institution’s Evidence Based Practice Website, adapted for internal use from Leung et al.^[10]^ CTPA = computed tomography of the pulmonary artery, CXR = chest radiograph, LE Doppler = lower extremity Doppler, PE = pulmonary embolism, V/Q = ventilation perfusion scan.

This retrospective blinded reader study was approved by the Institutional Review Board. All VQ scans performed on pregnant patients between March 2012 and July 2017 at the associated hospitals were collated. Consent was waived as this was a retrospective imaging review. The clinical algorithm for imaging pregnant patients during the review period is given in Figure 1.^[10]^ This standard imaging protocol included both ventilation imaging (bilateral posterior oblique dynamic 133Xenon gas [5–30 millicuries (mCi)] images and perfusion imaging (6 standard planar views (frontal/posterior, bilateral oblique) after injection of ^99m^Tc-MAA (1 mCi).

Images were retrieved from PACS and anonymized in a standard image viewing platform, MIM (Cleveland, OH). Images from each patient were presented twice to each reader: once with both ventilation and perfusion images (VQ), and once with perfusion images (Q) without ventilation images. The correlative X-ray was included with each set.

Eight readers participated in this study, including 4 nuclear radiology trainees (3 nuclear radiology fellows that completed radiology residency and 1 integrated nuclear radiology fellow in the final year of radiology residency) and 4 board-certified nuclear medicine physicians with varying years of experience (3, 7, 10, and 15 years). The VQ and Q images were presented to each reader in a random order. Readers were instructed to read the cases in an assigned order over 8 days. Daily sessions consisting of VQ and Q images (each 50%) were assigned, ensuring that the VQ and Q images from the same study would not be viewed on the same day. Readers recorded each study as PE, no PE, or non-diagnostic through a web portal emulating a clinical read provided in routine clinical care. Readers were instructed to read each case only once and not to alter any response retrospectively.

An intrareader data set of 24 cases read as duplicate VQ and Q scans was also integrated into the workflow to study the reproducibility of a reader to classify scans.

### 2.2. Statistics

All statistical analyses will be conducted in the R software environment or Stata/IC 15.0 for Mac (StataCorp, College Station, TX) with 2-sided hypothesis tests and assuming a 5% type I error rate. Since a standard of truth was not established for each study (i.e. positive and negative studies were not confirmed through pulmonary angiography, CT angiography, clinical follow-up or any other method), diagnostic performance statistics such as test sensitivity, specificity, negative predictive value, and positive predictive value were not calculated.

The median number of positive/negative/non-diagnostic studies was calculated for each type of study and by reader qualifications. Differences in the median percentages between types of studies and types of readers were calculated using a 2-sample test of proportions.

Logistic mixed effect models with random intercepts for subjects and readers were used to determine an association between scan type and rating in positive versus negative studies, positive versus indeterminate studies, and negative versus indeterminate studies (see supplemental material for model details). To examine scan type effect for intra-rater, a small sample of studies (n = 24) was read twice in each presentation (twice as VQ scans and twice as Q scans) and a logistic mixed model was used to determine an association between scan type and rating.

Fleiss kappas were performed to examine the agreement among faculty and fellows and their confidence intervals were reported. A 2-sided *z*-test was used to test differences in these metrics. Lastly, individual kappa values were calculated to quantify the agreement between V and VQ scans for each reader.

## 3. Results

Two hundred and 3 pairs of studies in 197 patients were included (6 patients had 2 scans). Administered activity was available for 168 studies: median of 8.9 mCi (range 4.3–23.6 mCi) for inhaled ^133^Xenon gas and median of 1.1 mCi (range 0.9–4 mCi) for intravenously injected ^99m^Tc-MAA. No adverse events were reported. Subjects ranged from 14 to 45 years of age, with a median of 28 years.

Summary statistics for the classification of the studies are given in Table 1, also stratified by reader qualifications (attending and fellows). No data was missing. As expected, the rate of positive tests was quite low, with a median less than 9% by any metric. The median proportion of positive studies and negative studies was not different between types of studies among a type of readers (VQ vs Q for fellows or attendings) or between types of readers of the same study (VQ for fellows/residents vs Q for fellows/residents). The proportion of non-diagnostic tests were not different between VQ versus Q scans for attendings or for fellows. However, fellows did classify more scans as non-diagnostic for both VQ and Q than attendings. Fellows deemed 4.4% and 6.4% of VQ and Q scans, respectively, as non-diagnostic while attendings classified only 0.2% of each type of scan as non-diagnostic.

**Table 1:** Median (percentage/range when applicable) number of studies in each classification by reader type.

	Fellows		Attendings		All	
	VQ	Q	VQ	Q	VQ	Q
Positive	13 (6.4%)	12.5 (6.2%)	13.5 (6.7%)	17 (8.4%)	13.5 (6.7%) (range: 8–15)	15.5 (7.6%) (range: 10–18)
Negative	180 (88.7%)	176.5 (86.9%)	188.5 (92.9%)	185.5 (91.4%)	188 (92.6%) (range: 176–192)	184.5 (90.9%) (range: 173–191)
Non-diagnostic	9 (4.4%)	13 (6.4%)	0.5 (0.2%)	0.5 (0.2%)	2.5 (1.2%) (range: 0–15)	1.5 (0.7%) (range:0–16)

Q = perfusion only scan, VQ = ventilation perfusion scan pair.

Logistic mixed effects models were used to test the association between scan type (VQ vs Q) versus ratings (tested in pairs: positive vs negative; positive vs non-diagnostic; negative vs non-diagnostic). There was a significant association between scan type and positive/negative classification (*P*-value of 0.0488). Q scans received more positive classifications than VQ scans. No association was seen between scan type and positive/non-diagnostic classification, nor between scan type and negative/non-diagnostic classification (*P*-value of 0.794 and 0.291, respectively).

Analysis of the intrareader data set (24 cases read as duplicate VQ scans and Q scans) did not reveal an association between scan type and classification. This result, though, should be interpreted with caution as the sample size was small and consequently the result is underpowered.

Agreement among faculty and attendings was then studied. We again note that the ground truth for each study was not established. The Fleiss’ kappas for faculty and fellows reading VQ scans were 0.646 (95% CI: [0.602, 0.690]) and 0.376 (95% CI: [0.332, 0.420]) respectively, with a significant difference achieved (*P*-value < 0.001). The results indicated the agreement of the faculty was significantly higher than that of the fellows. Similarly, for Q scans, the Fleiss’ kappa for faculty and fellows were 0.635 (95% CI: [0.587, 0.682]) and 0.473 (95% CI: [0.429, 0.517]) respectively, with a significant difference achieved (*P* < .001). This, too, demonstrated better agreement among the faculty compared to the fellows.

**Figure 2.**
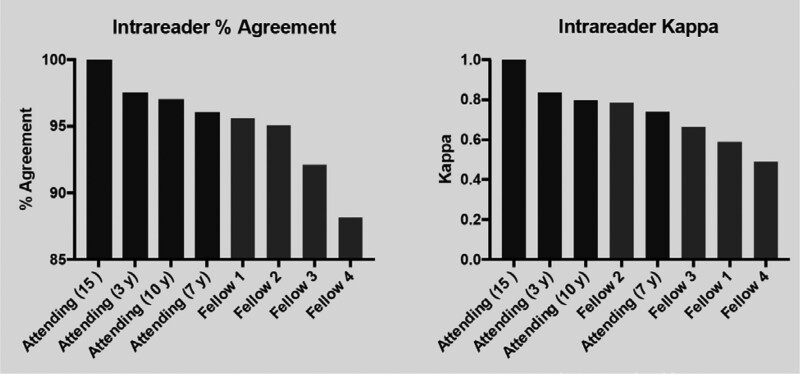
Agreement among each individual reader for a particular scan pair (ventilation-perfusion and perfusion only). The left bar graph demonstrates the % intrareader agreement. The right bar graph shows the kappa, which is used to test reader reliability. The attending with the most years of experience demonstrating the best reliability, and the fellows generally demonstrating lower reliability.

Agreement among each individual for a particular scan is shown in Figure 2. One attending, the one with the most experience (15 years), had 100% agreement for a kappa of 1.0. In general, attendings had better agreement than the fellows.

## 4. Discussion

In this retrospective reader study, perfusion-only imaging was associated with more positive interpretations than ventilation-perfusion imaging when analyzed with a logistic mixed effect model. The absolute increase in positive studies, though, was quite small: a median of 13.5 studies were classified as positive when ventilation and perfusion images were available compared to a median of 15.5 studies when only perfusion imaging was utilized. With 203 exams in this study, this represents a difference in medians of less than 1%. Thus, although a statistical difference in the rate of positivity between the scan types was achieved, likely secondary to a large sample size, the magnitude of difference suggests that both imaging methods are similar in their overall performance. Given the benefits of omitting ventilation imaging – particularly in the setting of the recent pandemic – perfusion-only imaging should be considered a reasonable option for imaging of a pregnant patient.

Although pregnant patients are at increased risk for PE, this patient population is relatively young (median age of 28 in this study) and usually healthy. Scans are overwhelmingly negative (greater than 90% in our series), in part owing to physiologic changes of pregnancy mimicking the symptoms of acute PE and a low clinical threshold for further evaluation of this at-risk population. These findings corroborate that of other research groups which demonstrated that the diagnostic yield in VQ scan of pregnant patients was much higher (73%–92%) due to the high percent of normal exams and low number of non-diagnostic exams.^[11]^ This highly contrasts the original PIOPED study in which only 14% of studies were deemed near normal/normal.^[12]^

**Figure 3.**
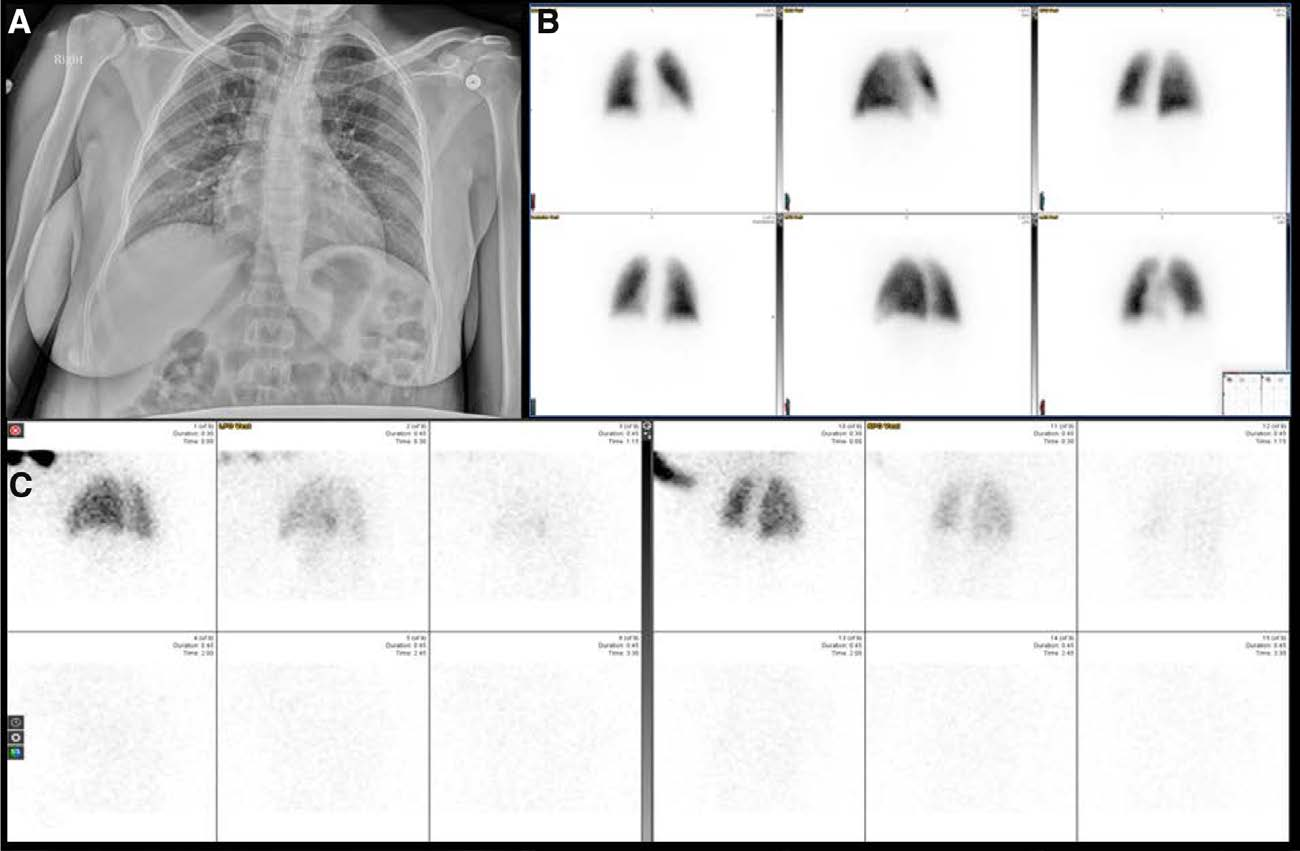
Example of a normal scan with both ventilation-perfusion and perfusion only imaging interpreted as pulmonary embolism absent by all readers. (A) Same day chest radiograph; (B) perfusion imaging with ^99m^Tc-Macroaggregated albumin; (C) ventilation imaging with ^133^Xenon.

**Figure 4.**
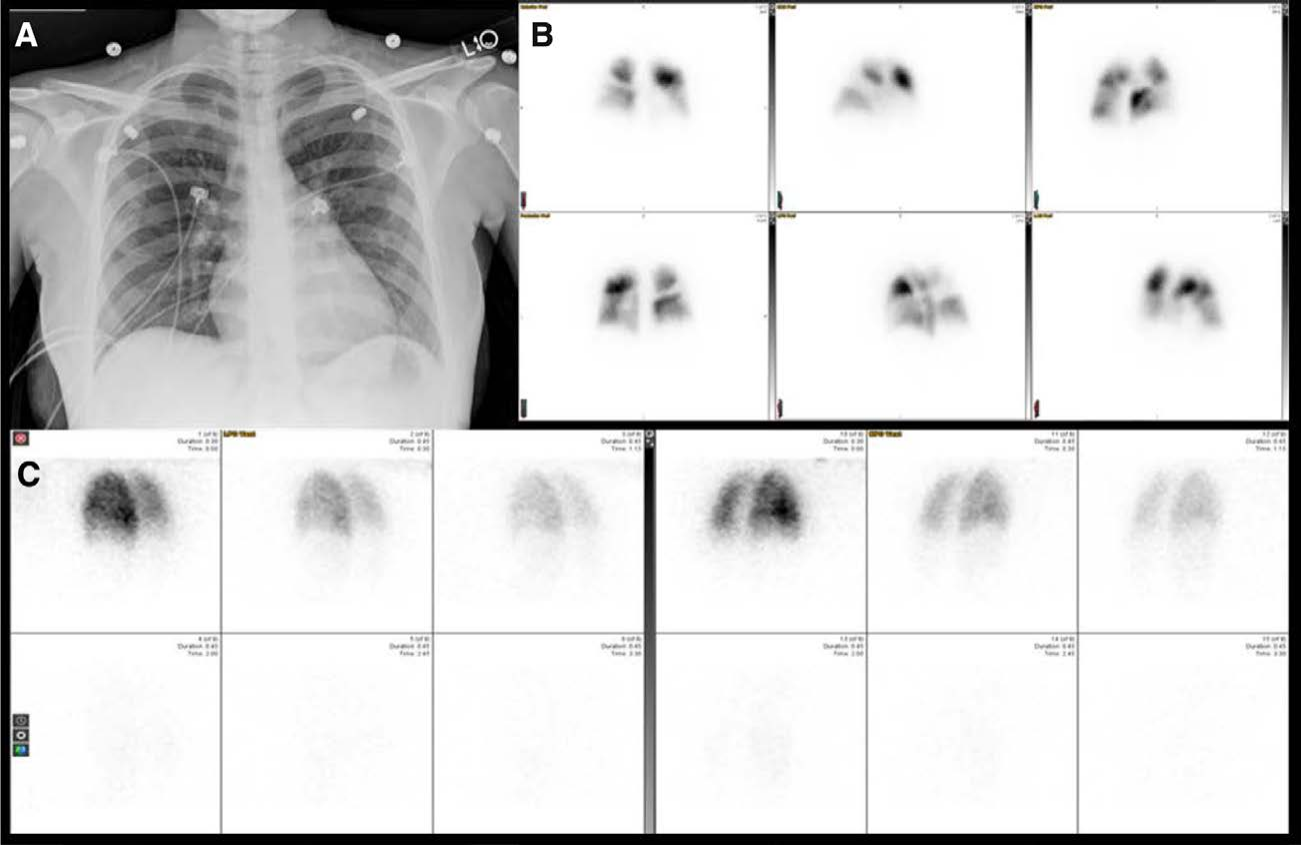
Example of an abnormal scan with both ventilation-perfusion and perfusion only imaging interpreted as pulmonary embolism present by all readers. (A) Same day chest radiograph; (B) perfusion imaging with ^99m^Tc-Macroaggregated albumin; (C) ventilation imaging with ^133^Xenon.

**Figure 5.**
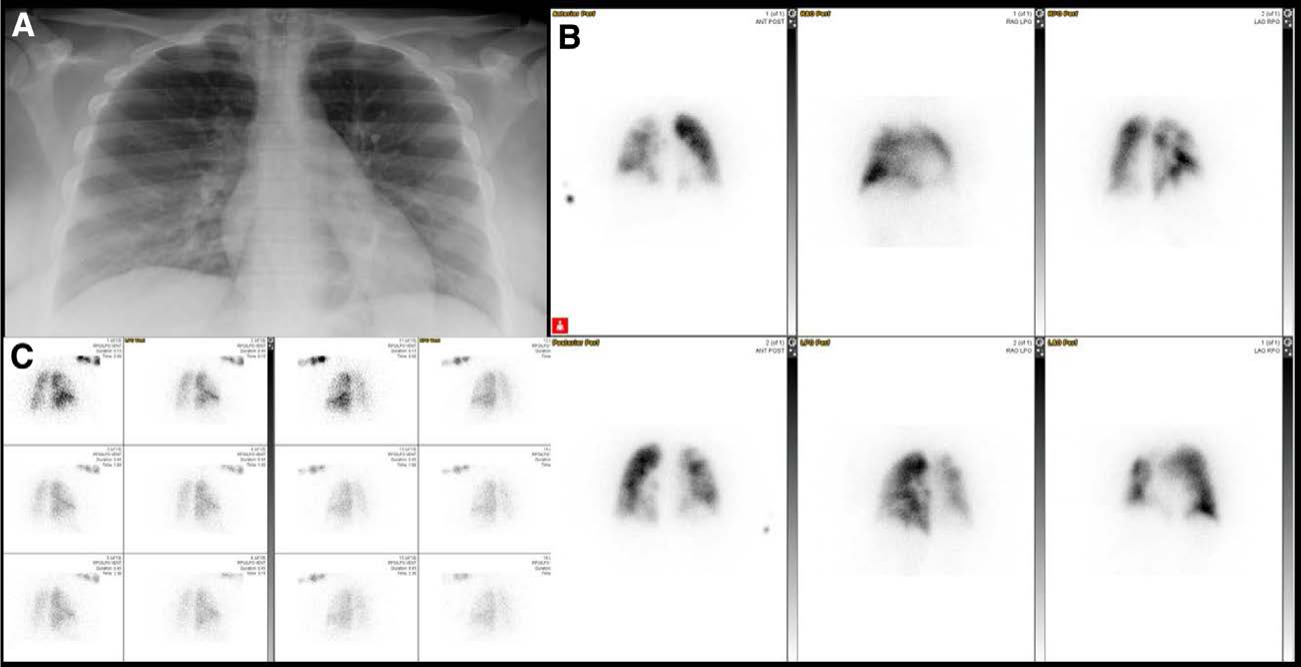
Example of an abnormal scan where the presence of ventilation altered readers’ interpretation with several changing the diagnosis from pulmonary embolism present to nondiagnostic. (A) Same day chest radiograph; (B) perfusion imaging with ^99m^Tc-Macroaggregated albumin; (C) ventilation imaging with ^133^Xenon.

In this predominately healthy population, very few patients had complicating respiratory comorbidities in which the ventilation images would possibly help in excluding other etiologies. Consequently, ventilation images were overwhelmingly negative, and not contributory (Figs. 3 and 4). For those few patients where the presence of the ventilation altered scan interpretation (Fig. 5), the VQ study was judged negative or non-diagnostic while the Q study was deemed positive. Indeed, a statistically significant increase in the rate of positive interpretation in Q studies compared to VQ studies was found (*P*-value of 0.0488). This is expected given the addition of ventilation imaging improves the specificity of the exam for pulmonary emboli. In the prenatal period, many different pulmonary parenchymal conditions may affect the scintigraphic appearance of the lung, including, pneumonia, aspiration, asthma, air trapping, and mucus plugging. Such abnormalities would lead to a matched defect if both ventilation and perfusion imaging was obtained, but may be interpreted as a PE in the absence of ventilation imaging (Fig. 5). The absolute number of the 203 scans affected by a change in their interpretation when V was added to the interpretation was extremely small. Depending on the interpreter, the number of affected patients ranged from –2 (representing 2 more studies classified as positive with VQ compared to Q) to 6 (6 more studies classified as positive on Q imaging compared to VQ), with the most common value being 2. This represents between 0% and 3% of patients in the study group, underscoring the minimal impact ventilation has on the interpretation of images for this patient population.

Overall, there was only the rare exam that was interpreted as non-diagnostic. The median of non-diagnostic for VQ scans for all reader was 2.5, or 1.2% of all exams. In addition, the analysis did not demonstrate an association between non-diagnostic scans and either positive or negative scans. This low percent of non-diagnostic exams at our institution may be attributed to the consistent use of an imaging algorithm that diverts women with leg symptoms or abnormal CXRs to another form of imaging (primarily lower extremity Doppler or chest CTPA, respectively). By implementing this algorithm (Fig. 1), many non-diagnostic exams may have been averted, while still maintaining appropriate patient care. Specifically, with regard to the objectives studied here, identifying patients with asthma/Chronic Obstructive Pulmonary Disease to image with chest CTPA would theoretically decrease the prevalence of patients with abnormal ventilation scans in our study. Ultimately, this selection factor may mitigate performance differences between VQ and Q scans. In fact, in at least one study looking at VQ scans in pregnant patients, as many as half of the exams interpreted as non-diagnostic, should have been evaluated by other means (e.g., CTPA) as indicated by the pre-imaging clinical findings, and highlights the importance of implementing pre-imaging algorithms.^[7]^ This also suggests a note of caution in translating the findings of this study to unscreened populations. While the overall number of non-diagnostic exams in our prescreen population were low, it is also reassuring for ordering clinicians to know that in a retrospective study evaluating the outcomes in pregnant patients who underwent VQ scanning, that there was no reported development of clinically evident PE or venous thromboembolism in those reported as non-diagnostic.^[11,13]^

Agreement between the 4 attendings physicians was significantly better than that of the 4 fellows for both V scans and VQ scans (0.635 vs 0.473, *P*-value < 0.001). In general, intrareader agreement was also greater with attending physicians than fellows (0.646 vs 0.376, *P*-value < 0.001). These differences are not surprising given differences in experience in reading these scans (ranging from 1 year in the fellows to 15 years in the most experienced attending). Other investigators have shown that an experienced nuclear radiology physician can accurately gestalt the conclusion of a VQ scan based on experience.^[14]^ With much less experience, it is not unexpected that nuclear radiology fellows have less diagnostic agreement.^[12,15]^

There are several limitations to this retrospective study. Although retrospective, the number of readers (8) and cases (203) compares favorably to prior studies.^[11,13]^ No ground truth was established for each study, precluding the evaluation of sensitivity, specificity, etc. No specific interpretation criteria were recommended, though the discrete classifications used (PE present, PE absent, and non-diagnostic) are the same as those used in the prospective investigative study of acute PE diagnosis criteria and in the Perfusion only Modified PIOPED II criteria.^[16]^ Moreover, these classifications are the same as those rendered on these studies during routine clinical care at our institution, underscoring the clinical relevance of this study. Readers also were instructed to complete the assigned cases on sequential days, but compliance, as expected, was not perfect. The careful selection of subjects in this study – pregnant patients with normal CXRs – also tempers widespread application of these conclusions, as discussed above.

The exercise of sequentially reading these studies received positive evaluations from the fellows regarding its educational value, although, this study was not designed to rigorously test this method as a learning tool. In particular, the fellows commented that they felt more comfortable in interpreting the studies as they gained experience. Indeed, for all participating fellows, participation in this study more than quadrupled the sum of Q and VQ studies interpreted, with the range of clinical exams (both VQ and Q) interpreted by the fellows during their training year at 37 to 57. For future studies, agreement with a reference standard (possibly a selected attending, perhaps the most experienced attending) may be calculated per day to demonstrate improvement in interpretation, as well as possibly identify a point of diminishing returns for trainees. While there is currently no data on the topic of what quantifies an expert in the interpretation of imaging, there has been a recent call for an examination of the nature of imaging expertise and for rigorous scientific examination of educational tools for use in radiology education and training.^[17,18]^

## 5. Conclusion

In this study, we demonstrated that the exclusion of ventilation images in VQ scans was associated with a higher rate of positive studies, but the overall number of studies affected was quite small and felt not to be clinically significant. In fact, pulmonary perfusion imaging of pregnant patients at our institution is now performed with this perfusion only protocol. Similarly, ventilation imaging has been eliminated in the evaluation of non-pregnant patients suspected of having a PE. There are ongoing investigations into which test is best for the non-pregnant patient since the onset of the pandemic. In the non-pregnant patient population, perfusion-only Single Photon Emission Computed Tomography (SPECT)/CT is now frequently performed, with anatomic changes in the lung parenchyma on CT serving as a proxy for ventilation images. In this setting, one study demonstrated that SPECT/CT increases sensitivity, but slightly degrades specificity.^[19]^ Given radiation safety considerations, CT is unlikely to be added to the imaging protocol for pregnant women, especially in a younger patient population with normal CXRs. Other practices have eliminated ventilation imaging in favor of perfusion-only planar images with acceptable performance as a screening test.^[20]^ We favor perfusion-only planar imaging over SPECT imaging for pregnant patients since CT images are typically not available for comparison, planar image acquisition is faster, and there is the potential for suboptimal image quality with SPECT reconstructions given the adjusted low dose administered to this patient population. This study supports such a practice change.

## Author contributions

**Conceptualization:** Vlasios S. Sotirchos, Andrew Kozlov, Katrina Korhonen, Daniel A. Pryma.

**Data curation:** Andrew Kozlov, Daniel A. Pryma.

**Formal analysis:** Jennifer A. Schroeder, Quy Cao, Jennifer A. Gillman.

**Investigation:** Jennifer A. Schroeder, Vlasios S. Sotirchos, Jennifer A. Gillman, Thomas Anderson, Stamatoula Pilati, Jacob G. Dubroff, Michael Farwell, Daniel A. Pryma.

**Methodology:** Katrina Korhonen.

**Project administration:** Vlasios S. Sotirchos, Katrina Korhonen, Daniel A. Pryma, Austin R. Pantel.

**Supervision:** Katrina Korhonen.

**Writing – original draft:** Jennifer A. Schroeder, Austin R. Pantel.

**Writing – review & editing:** Jennifer A. Schroeder, Quy Cao, Jennifer A. Gillman, Thomas Anderson, Stamatoula Pilati, Jacob G. Dubroff, Michael Farwell, Andrew Kozlov, Katrina Korhonen, Daniel A. Pryma, Austin R. Pantel.
